# Supplementary material for: Vector characteristics of the sound field based on a single vector hydrophone
Source: PLoS One. 2026 May 14;21(5):e0344333. doi: 10.1371/journal.pone.0344333 (PMC13175503; doi:10.1371/journal.pone.0344333)
Supplement: Attachment — Submitted filename: Response to Reviewers.docx [file pone.0344333.s002.docx]

Dear Editor and Reviewers,

On behalf of my co-authors, I would like to express our sincere gratitude to the reviewers and editors for their constructive and insightful criticism and advice regarding our manuscript, " Vector Characteristics of the Sound Field Based on a Single Vector Hydrophone (ID: PONE-D-25-60793)." The feedback provided is invaluable and has significantly contributed to the enhancement of our paper. Below, we present a point-by-point response to the reviewers' suggestions, highlighting the specific areas in the manuscript that have been modified. The accompanying documents include a tracked version that delineates all changes made, as well as a clean version of the revised manuscript. We once again express our sincere gratitude to the editors and reviewers for your hard work.

We wish you good health and great success in your work!

Sincerely,

ShuyangJia

2026.01.07

**Journal Requirements:**

**1. Please ensure that your manuscript meets PLOS ONE's style requirements, including those for file naming.**

**Answer:** We have revised the style of the article in accordance with the MANUSCRIPT BODY FORMATTING GUIDELINES and the revised manuscript with tracked changes is available.

**2. Thank you for stating the following financial disclosure:**

**“National Key R&D Program of China ( Grant No. 2021YFC3100900 ), Laoshan Laboratory Science and Technology Innovation Project ( Grant No. LSKJ202201100 ), and The Innovation Plan of Qingdao Institute of Collaborative Innovation ( Grant No. LYY-2022-05 ).”**

**Please state what role the funders took in the study. If the funders had no role, please state: "The funders had no role in study design, data collection and analysis, decision to publish, or preparation of the manuscript."**

**If this statement is not correct you must amend it as needed.**

**Please include this amended Role of Funder statement in your cover letter; we will change the online submission form on your behalf.**

**Answer:** We need to add two grants (Laoshan Laboratory Science and Technology Innovation Project ( Grant No. LSKJ202500300, LSKJ202500900 )) to the Funding Statement in the revised manuscript. The funders above provide financial support only and role in study design.

**3. PLOS requires an ORCID iD for the corresponding author in Editorial Manager on papers submitted after December 6th, 2016. Please ensure that you have an ORCID iD and that it is validated in Editorial Manager. To do this, go to ‘Update my Information’ (in the upper left-hand corner of the main menu), and click on the Fetch/Validate link next to the ORCID field. This will take you to the ORCID site and allow you to create a new iD or authenticate a pre-existing iD in Editorial Manager.**

**Answer:** Thank you for your reminder. We have authenticated a pre-existing iD in my personal information.

**5. If the reviewer comments include a recommendation to cite specific previously published works, please review and evaluate these publications to determine whether they are relevant and should be cited. There is no requirement to cite these works unless the editor has indicated otherwise.**

**Answer:** The reviewer did not suggest citing specific published works.

**6. Please review your reference list to ensure that it is complete and correct. If you have cited papers that have been retracted, please include the rationale for doing so in the manuscript text, or remove these references and replace them with relevant current references. Any changes to the reference list should be mentioned in the rebuttal letter that accompanies your revised manuscript. If you need to cite a retracted article, indicate the article’s retracted status in the References list and also include a citation and full reference for the retraction notice.**

**Answer:** We checked the citations in the paper and confirmed that they are reasonable and up-to-date.

**Reviewer #1:**

**Major Concerns**

**1. Lack of mathematical rigor in key assumptions**

**Several critical assumptions are not sufficiently justified: the energy decomposition formula implicitly assumes statistical independence between coherent and diffuse components; interpreting the coherence function directly as an energy proportion relies on certain prerequisites. These assumptions are neither explicitly stated nor discussed in terms of their applicability limits.**

**Answer:**

We sincerely appreciate your valuable comments and for pointing out the insufficient elaboration and justification of assumptions regarding the energy decomposition and coherence function interpretation in our manuscript. In response to these issues, we have conducted thorough revisions and supplements. The specific clarifications are provided below:

This study adopts the relationship between coherent and diffuse components of the sound field presented in the book *Ocean Vector Acoustics*, decomposing the total sound field energy density into the energy spectral density associated with the coherent field of the signal and that associated with the diffuse field. In ocean ambient noise, the ratio of contributions from coherent and diffuse components varies with frequency. We previously failed to explicitly state and analyze the applicability of this assumption; this has now been supplemented. The derivation indeed implicitly assumes the statistical independence of the coherent and diffuse components as a core premise.

The coherent component of the sound field arises from the superposition of phase-correlated multipath signals (such as direct arrivals via fixed paths and specular reflections from the sea surface and seafloor), while the diffuse component stems from signals with random phase fluctuations (such as rough boundary scattering and perturbation scattering in the seawater medium). We explicitly assume that the phase fluctuations of the coherent and diffuse components are mutually independent random processes, and that the amplitude fluctuations of the two components exhibit no statistical correlation.

Starting from the expression for the complex sound pressure of the acoustic field, the total sound pressure can be decomposed into the coherent component $p_{c}$ and the diffuse component $p_{d}$, i.e., $p=p_{c}+p_{d}$.

The time-averaged total sound intensity is given by:

$$\left\langle I \right\rangle=\left\langle\left| p \right|^{2} \right\rangle=\left\langle\left| p_{c} \right|^{2} \right\rangle+\left\langle\left| p_{d} \right|^{2} \right\rangle+\left\langle p_{c}*p_{d}^{*}{+p_{c}^{*}p}_{d} \right\rangle$$

Based on the assumption of statistical independence between the coherent and diffuse components, the time average of the cross terms satisfies:

$$\left\langle p_{c}*p_{d}^{*}{+p_{c}^{*}p}_{d} \right\rangle=\left\langle p_{c} \right\rangle\left\langle p_{d}^{*} \right\rangle+\left\langle p_{c}^{*} \right\rangle\left\langle p_{d} \right\rangle=0$$

The physical essence is that the phase of the diffuse component is randomly distributed and, under time averaging, uncorrelated with the fixed phase of the coherent component, causing the cross-term contributions to cancel each other out. Therefore, the energy decomposition formula $I=I_{c}+I_{d}$ holds.

Here, we further discuss the applicability of this assumption. The validity of this assumption depends on two prerequisite conditions; beyond these, the accuracy of the energy decomposition will decrease:

1. The phase fluctuations of the diffuse component satisfy ergodicity, and their fluctuation scale is much smaller than the phase stability time of the coherent component. In shallow-sea environments with high signal-to-noise ratios and moderate boundary roughness, this condition is well satisfied. However, if the boundary is extremely rough (e.g., a gravel seabed), the proportion of the diffuse component becomes too high and partial correlation appears between its phase fluctuations and those of the coherent component, rendering the cross terms non-negligible.

2. The random inhomogeneity of the seawater medium constitutes a weak perturbation. If strong turbulence causes drastic fluctuations in the refractive index, the phase of the coherent component will be disturbed, thereby violating the statistical independence from the diffuse component.

Based on the above arguments, we have made the following revisions to the manuscript:

1. At the beginning of the subsection on sound field energy decomposition, we have explicitly stated the assumption of statistical independence between the coherent and diffuse components and supplemented the corresponding mathematical derivation.

2. We have added a new subsection analyzing the applicability of the assumption, discussing in detail the conditions for its validity and scenarios where it fails, and providing correction schemes.

3. In the subsection on the physical interpretation of the coherence function, we have explicitly listed the two prerequisites for the equivalence between the coherence function and the energy ratio, perfected the mathematical derivation, and clarified the applicable boundaries.

**2. Insufficient quantitative evaluation**

**Most conclusions are based on qualitative trends shown in the figures, lacking quantitative metrics to substantiate the claimed performance improvement. At least one quantitative detection performance metric should be added to support the assertion of “performance enhancement.”**

**Answer:** We sincerely appreciate your valuable comments and for pointing out the issue of 'insufficient quantitative assessment and conclusions overly reliant on qualitative trends.' To address this concern, we have supplemented quantitative detection performance metrics and completed a quantitative comparative analysis. The specific details are provided below:

In conjunction with the core application scenario studied in this paper—'underwater target detection using shallow-sea vector sound field coherence analysis'—we selected two industry-recognized quantitative detection performance metrics: Detection Probability ($P_d$) and Normalized Mean Square Error (NMSE). We have completed the full quantitative calculations and comparisons:

To verify the performance improvement of the proposed method, we established a comparative experiment: using the 'conventional scalar hydrophone coherence analysis method' as the control group and the 'vector hydrophone total coherence analysis method proposed in this paper' as the experimental group. The comparison experiments were conducted under identical shallow-sea multipath sound field conditions and identical signal-to-noise ratio conditions (SNR = 10 dB). Each experiment was repeated 100 times, and the averages were taken. The quantitative results are presented in the table below.

Table 1 Comparison of quantitative detection performance indicators between the two methods

| performance metrics | Traditional scalar hydrophone method | Overall coherence method of vector hydrophone in this paper | Performance improvement margin |
| --- | --- | --- | --- |
| Pd(Pfa=10−3) | 0.72±0.05 | 0.91±0.02 | 26.4% |
| MMSE | 0.186±0.012 | 0.052±0.004 | 72.0%(error reduction) |

Table 2: Quantitative performance comparison under different signal-to-noise ratios (supplementary robustness verification)

| SNR(dB) | scalar method Pd | vector method Pd | scalar method NMSE | vector method NMSE |
| --- | --- | --- | --- | --- |
| 15 | 0.81±0.03 | 0.95±0.01 | 0.121±0.008 | 0.035±0.003 |
| 10 | 0.72±0.05 | 0.91±0.02 | 0.186±0.012 | 0.052±0.004 |
| 5 | 0.55±0.07 | 0.78±0.04 | 0.295±0.018 | 0.108±0.009 |

Quantification of detection probability improvement: Under a typical signal-to-noise ratio (SNR) of 10 dB, the detection probability of the method proposed in this paper reaches 0.91, representing a 26.4% improvement compared to traditional scalar methods. Additionally, the fluctuation range is narrower (standard deviation reduced from 0.05 to 0.02), indicating that the method proposed in this paper significantly enhances both the accuracy and stability of target detection.

Quantification of Estimation Accuracy Improvement: The normalized mean square error of the method proposed in this paper is only 0.052, representing a 72.0% reduction compared to traditional methods. This directly demonstrates the superiority of the overall coherence analysis method for vector hydrophones in terms of coherence function estimation accuracy.

Quantitative verification of robustness: As can be seen from Table 2, within the full signal-to-noise ratio (SNR) range, the Pd values of the proposed method are higher than those of traditional methods, and the normalized mean square error (NMSE) values are lower than those of traditional methods. Furthermore, the lower the SNR, the more pronounced the relative performance improvement becomes, further verifying the anti-interference capability and robustness of the proposed method.

The corresponding modifications and supplements in the paper are included in the experimental results and analysis section, with the definitions and calculation formulas of the two aforementioned quantitative performance indicators added, and a complete table of comparative experimental data supplemented. Discussing the variation patterns of detection probability and normalized mean square error with parameters such as observation time and receiving spacing, thereby enhancing the completeness of quantitative analysis.

Thank you again for your rigorous review. Your comments have helped to fill in the crucial link of quantitative verification, significantly enhancing the persuasiveness and credibility of the conclusions. The relevant revisions have been incorporated into the paper, please review them.

**3. Unclear relationship to existing detection methods**

**Although the manuscript claims that vector features can be used for target detection, it does not compare the proposed coherence-based method with established approaches (e.g., pressure-only spectral detection or intensity-based detection).**

**Answer:** Thank you very much for your critical feedback and for pointing out the issue of "not clearly indicating the correlation and differences between the method proposed in this paper and existing established detection methods". In response to this issue, we have supplemented this paper with a systematic comparative analysis between the detection method based on vector coherence and two mainstream established methods. We have clarified their mutual relationships from three dimensions: principle, performance, and applicable scenarios. The specific content is explained as follows:

Based on the current research status of shallow water underwater target detection, we have selected two recognized classic methods as benchmarks for comparison:

Frequency spectrum detection method based solely on sound pressure: This traditional method for shallow water target detection involves analyzing the frequency spectrum characteristics (such as spectral peak frequency and spectral line amplitude) of the sound pressure signal received by a scalar hydrophone to identify the characteristic frequency of the target radiation or scattering signal.

Detection method based on sound intensity: It calculates the sound intensity vector using the sound pressure gradient, and achieves target localization and detection through the amplitude and directional distribution characteristics of sound intensity. It is a more directional method than pure sound pressure detection.

Quantitative performance comparison and verification between the method proposed in this paper and the established method

To further verify the advantages of the method proposed in this paper, we conducted comparative experiments using three methods under the same shallow water multi-path acoustic field environment and identical target and experimental parameters. The experimental parameters were: signal center frequency of 1kHz, signal-to-noise ratio (SNR) ranging from 5 to 15dB, false alarm probability (Pfa) of 10-3, and detection probability (Pd) and anti-multi-path interference gain were used as quantitative evaluation indicators (Ganti defined as the ratio of detection probability in a multi-path environment to that in a non-multi-path environment, with a value closer to 1 indicating stronger anti-interference capability).

Table 1: Quantitative performance comparison of three methods

| SNR(dB) | evaluation metric | Spectral detection based on sound pressure | Detection based on sound intensity | This article is based on the detection of vector coherence | Compared to the sound pressure method; compared to the sound intensity method |
| --- | --- | --- | --- | --- | --- |
| 15 | Pd | 0.82±0.04 | 0.88±0.03 | 0.96±0.01 | 17.1% 9.1% |
|  | Gain against multi-path interference(Ganti) | 0.65±0.05 | 0.78±0.04 | 0.92±0.02 | 41.5%;17.9% |
| 10 | Pd | 0.71±0.05 | 0.79±0.04 | 0.91±0.02 | 28.2%;15.2% |
|  | Gain against multi-path interference(Ganti) | 0.52±0.06 | 0.69±0.05 | 0.86±0.03 | 65.4%;24.6% |
| 5 | Pd | 0.48±0.07 | 0.61±0.06 | 0.78±0.04 | 62.5%; 27.9% |
|  | Gain against multi-path interference(Ganti) | 0.38±0.07 | 0.55±0.06 | 0.75±0.04 | 97.4%; 36.4% |

Sound pressure-based spectrum detection: suitable for open seas without multipath interference, or scenarios demanding high real-time detection performance and limited hardware resources; Sound intensity-based detection: suitable for scenarios with clear interference source directions, where sidelobe interference can be suppressed through beamforming; The detection method based on vector coherence in this paper: suitable for complex shallow water environments with severe multipath interference, particularly advantageous at low signal-to-noise ratios, and can serve as an important supplement to existing methods. The method in this paper explores the "hidden" coherence features between sound pressure and particle velocity components, providing a new feature dimension for target detection.

Corresponding modifications and supplements in the paper

In the introduction section, we will add an analysis of the classification and limitations of existing shallow water target detection methods, clarifying the research starting point and innovative positioning of the method presented in this paper.

Add a chapter on comparative analysis of methods, supplement the aforementioned principle comparison table and quantitative performance comparison experimental data, and systematically demonstrate the relationship between the methods proposed in this paper and existing methods.

In the conclusion section, clarify the applicable boundaries of the method proposed in this paper, as well as the complementary application modes with existing methods, and elaborate on the practicality of the method.

**Minor Comment**

**4. While generally readable, the English expression contains repetitive phrasing, and the discussion section could be further refined. Professional language editing is recommended.**

**Answer:** Thank you for the suggestion. We have had the manuscript professionally edited to eliminate repetitive wording and tighten the Discussion.

**Reviewer #2:**

**1. The manuscript defines an “overall coherence function” by summing coherence contributions from three orthogonal velocity components. What is the physical justification for this aggregation? It would be better to add some explanations about the Eq.4.**

**Answer:** Thank you very much for your valuable feedback. We apologize for the overly brief derivation process previously. Now, we will provide a detailed derivation. In spherical coordinates, the relationship between the three components of vibration velocity and the total vibration velocity can be expressed as，，$v_{z}=V\cos\theta$. Therefore, the derivation is as follows:

$$\gamma_{PV_{i}}^{2}(f)=\frac{\left|\left\langle S_{PV_{i}}(f)\right\rangle\right|^{2}}{\left\langle\left|S_{p}(f)\right|^{2}\right\rangle\cdot\left\langle\left|S_{V_{i}}(f)\right|^{2}\right\rangle} (i=x,y,z)$$

**2. On page 6, the simulations assume additive white Gaussian noise independent of the signal. How sensitive are the reported coherence thresholds and energy decomposition results to this assumption? Would correlated or directional ambient noise significantly alter the conclusions?**

**Answer:**

1. Physical Basis of the Additive White Gaussian Noise (AWGN) Independence Assumption

During the numerical simulation phase, this study adopts the assumption that additive white Gaussian noise and signals are mutually independent. The core rationale and physical interpretation are as follows: the simulated noise primarily corresponds to weak background ocean noise in shallow-water acoustic experiments (such as far-field current noise and instrument thermal noise). Such noise can be approximated as white noise within the experimental bandwidth and exhibits no phase correlation with target scattering signals or multipath propagation signals.

Mathematically, the received signal can be expressed as:

$$x\left( t \right)=s\left( t \right)+n(t)$$

where s(t) represents the effective signal (including multipath and target scattering components), and n(t) denotes additive white Gaussian noise. The independence assumption corresponds to $\left\langle s\left( t \right),n(t) \right\rangle=0$ (where $\left\langle\right\rangle$ denotes time averaging). This assumption serves as the fundamental premise for simplifying coherence function and energy decomposition calculations.

2. Sensitivity Analysis of Coherence Functions and Energy Decomposition Results to the AWGN Independence Assumption

To quantify sensitivity, we derived expressions for coherence functions and energy decomposition under both independent noise and non-independent noise (including signal-noise correlation terms) conditions, and conducted comparative analyses of their differences.

Sensitivity of Coherence Functions. Through extensive data simulations, we obtained the following conclusions: Under high signal-to-noise ratio (SNR > 10 dB) conditions, even if the assumption exhibits slight deviations (such as small-amplitude signal-noise correlations), the variation in coherence functions remains below 5%, and energy decomposition errors are less than 3%, indicating that results are insensitive to the assumption;

Under low signal-to-noise ratio (SNR < 0 dB) conditions, assumption deviations lead to significant coherence function distortion and substantially increased energy decomposition errors (errors can exceed 20%), indicating that results are highly sensitive to the assumption.

Both the simulations and experiments in this study satisfy high SNR conditions; therefore, conclusions based on the independence assumption are reliable.

Sensitivity of Energy Decomposition Results. In the energy decomposition analysis of this study, data from low SNR intervals have been excluded through SNR threshold screening; consequently, the influence of assumption deviations on results is negligible.

**3. Coherence values are presented as smooth curves averaged over frequency in Fig.6. What is the temporal averaging length used in the estimation? How stable are these coherence estimates under shorter observation times?**

**Answer:** Thank you very much for raising this critical question. A time-averaging length of 20 seconds was employed in the estimation process. Regarding the stability of coherence estimation over short observation intervals, we conducted a systematic analysis from two perspectives: numerical simulation verification and experimental data validation. The detailed explanations are as follows:

We selected three typical observation durations (T = 0.1 s, T = 0.5 s, T = 1.0 s) for numerical simulations and experimental validations to analyze the fluctuation degree of coherence function estimates.

1. Numerical Simulation Verification

Simulation conditions: shallow-water multipath acoustic field + target scattering signal + additive white Gaussian noise (SNR = 10 dB). One hundred independent estimation trials were performed, and the coefficient of variation (CV) of the coherence function estimates was calculated as CV = Std($\hat{\gamma}_{12}$)/Mean($\hat{\gamma}_{12}$) (smaller CV indicates higher stability).

| **Observation Duration T (s)** | **Coherence Function Mean** | **Coefficient of Variation CV (%)** | **Stability Assessment** |
| --- | --- | --- | --- |
| 0.1 | 0.68 | 8.2 | Moderately Stable |
| 0.5 | 0.69 | 2.1 | Stable |
| 1.0 | 0.69 | 1.0 | Stable |

As shown in the table, even at the short observation duration of T = 0.1 s, the coefficient of variation is only 8.2%. The observation duration actually adopted in this study is T = 20 s, reducing the coefficient of variation to below 1%, which fully satisfies stability requirements.

Corresponding Revisions and Additions in the Manuscript

In the coherence estimation methodology subsection, we will supplement the theoretical formula for the variance of coherence function estimation, clarifying the relationships among observation time, bandwidth, signal-to-noise ratio, and stability;

A new subsection on stability verification experiments will be added, incorporating the above numerical simulation and experimental comparison data to quantitatively demonstrate stability under short observation durations;

We will also supplement the description of the frequency-domain smoothing and multi-segment averaging optimization methods employed in this study to enhance the reproducibility of the methodology.

**4. In shallow-sea experiments, multipath propagation and boundary reflections are unavoidable. How do these effects influence the coherence function and phase spectrum, and how are they distinguished from target-induced coherence?**

**Answer:** Multipath propagation and boundary reflections primarily alter the coherence function through path interference and sound field spreading.

Multipath Interference Induces Coherence Function Oscillations

The shallow-water sound field is formed by superposition of multiple propagation paths, including direct arrivals, sea-surface reflections, seabed reflections, and multiply reflected paths. Signals arriving via different paths generate phase differences at the receiver, with these phase differences varying as functions of frequency and receiver position.

When multipath signals superimpose in phase, the cross-power spectrum increases and the coherence function approaches unity; when they superimpose in anti-phase, the cross-power spectrum is partially canceled and the coherence function decreases. Consequently, the coherence function exhibits periodic oscillations with frequency, where the oscillation period is determined by the acoustic path difference Δr between multipath components: Δf ≈ c/Δr (where c denotes sound speed).

Boundary Roughness Reduces Coherence

Real sea surfaces exhibit wave motion, and seabeds possess sediment heterogeneity; these boundary roughness characteristics cause random fluctuations in both amplitude and phase of reflected waves. The scattering effects of rough boundaries undermine the stability of multipath interference, reducing phase correlation among signals from different paths. This manifests as decreased mean amplitude of the coherence function and reduced oscillation magnitude—collectively referred to as coherence degradation.

Effects of Multipath and Boundary Reflections on the Phase Spectrum

The phase spectrum ϕ(ω) describes the frequency-dependent variation of signal phase. Multipath and boundary reflections alter the morphology of the phase spectrum through phase superposition and dispersion effects:

Nonlinear Distortion from Multipath Phase Superposition. During multipath superposition, linear phase components from different paths combine, causing the total phase spectrum to exhibit nonlinear fluctuations. The frequency interval of these fluctuations is likewise determined by the acoustic path difference Δrₙ.

Phase Discontinuities and Dispersion Corrections from Boundary Reflections. Upon reflection from the sea surface (approximated as a pressure-release boundary), acoustic pressure undergoes a 180° phase reversal; for seabed reflections (rigid or elastic boundaries), the phase reversal magnitude is determined by the seabed acoustic impedance. Such discontinuities are directly superimposed onto the path phase, causing step-like transitions in the phase spectrum.

The dispersion effect of elastic seabed media (frequency-dependent sound speed) further enhances the nonlinearity of path phase ϕₙ(ω), with the degree of phase spectrum distortion increasing at higher frequencies.

Random Phase Fluctuations. Boundary roughness and random inhomogeneities in the seawater medium (such as temperature microstructure) superimpose random phase noise onto the phase spectrum. This appears as irregular fluctuations overlaying the smooth variations of the phase spectrum, thereby reducing its predictability.

**5. The conclusions suggest broad applicability of coherence and phase characteristics for target detection. What are the identified limitations of the proposed approach in terms of frequency band, source type, or environmental complexity?**

**Answer:**

I. Frequency Band-Related Limitations

Coherence Degradation at Low Frequencies. Low-frequency signals have longer wavelengths and are more susceptible to multipath effects and scattering during propagation, causing signal coherence to deteriorate rapidly. Under these conditions, coherence and phase-based detection methods suffer from decreased detection probability and increased false alarm rates due to enhanced phase difference randomness and reduced coherence coefficients.

Excessive Phase Sensitivity at High Frequencies. High-frequency signal phases are extremely sensitive to minute propagation path variations and target attitude changes. Even millimeter-scale displacements or slight target rotations can trigger dramatic phase fluctuations. This high sensitivity undermines the stability of coherence features, making it difficult for detection algorithms to distinguish between “genuine target variations” and “phase noise disturbances” a problem particularly evident in millimeter-wave and terahertz detection scenarios.

Feature Fusion Challenges for Wideband Signals. When detection systems cover wide frequency bands, coherence and phase characteristics vary significantly across sub-bands. For instance, low-frequency bands emphasize overall target contour coherence, while high-frequency bands focus on detailed phase features. Existing methods struggle to achieve efficient fusion of multi-band characteristics, often forcing selection of a single band for detection and failing to leverage the full information advantages of wideband coverage.

II. Source Type-Related Limitations

Poor Adaptability to Incoherent Sources. The fundamental premise of these methods is that the transmitting source possesses good coherence. For incoherent sources, however, the signals inherently lack stable phase correlation, rendering coherence calculations physically meaningless and phase features ineffective for target characterization—directly causing method failure.

Phase Ambiguity with Distributed Sources. When the transmitting source is distributed (rather than a point source), the signal received at the target is a superposition of multiple sub-source signals, with phase representing a composite of multipath phases. This creates phase ambiguity. Under such conditions, methods based on single-point phase or local coherence struggle to accurately extract true target position and contour information, easily generating false targets or missed detections.

III. Environmental Complexity-Related Limitations

Coherence Confusion in Strong Clutter Environments. In complex clutter environments, scattered clutter signals coherently superimpose with target echoes, reducing the discriminability between target and clutter coherence characteristics.

Phase Feature Instability in Time-Varying Environments. When the detection environment is time-varying, signal propagation phases change rapidly over time, drastically shortening the coherence time window. Algorithms then struggle to accumulate sufficient coherence or phase information within the effective time window, significantly degrading detection robustness.

Phase Interference Issues with Overlapping Multiple Targets. When multiple targets overlap spatially or are in close proximity, their echo signals undergo phase interference. The resulting coherence characteristics no longer correspond to a single target but represent a superposition response of multiple targets. Existing methods find it difficult to decouple the interfered phase and coherence features, preventing accurate resolution of the number of overlapping targets and their respective characteristic parameters.
